# Coronary Vessel Wall Contrast Enhancement Imaging as a Potential Direct Marker of Coronary Involvement: Integration of Findings From CAD and SLE Patients

**DOI:** 10.1016/j.jcmg.2014.03.012

**Published:** 2014-08

**Authors:** Niharika Varma, Rocio Hinojar, David D’Cruz, Eduardo Arroyo Ucar, Andreas Indermuehle, Sarah Peel, Gerald Greil, Nicholas Gaddum, Phil Chowienczyk, Eike Nagel, Rene M. Botnar, Valentina O. Puntmann

**Affiliations:** ∗ Cardiovascular Imaging Department, Division of Imaging Sciences and Biomedical Engineering, King’s College London, London, United Kingdom; † The Lupus Unit, Rayne’s Institute, King’s College London, London, United Kingdom; ‡ Department of Medical Physics and Bioengineering, Division of Imaging Sciences and Biomedical Engineering, King’s College London, London, United Kingdom; § Cardiovascular Division, King’s College London, London, United Kingdom

**Keywords:** cardiac magnetic resonance, contrast-to-noise ratio, coronary enhancement, vessel wall remodeling, CAD, coronary artery disease, CE, contrast enhancement, CMR, cardiac magnetic resonance, CNR, contrast to noise ratio, CV, cardiovascular, LGE, late gadolinium enhancement, LV, left ventricle, PWV, pulse wave velocity, SLE, systemic lupus erythematosus

## Abstract

**Objectives:**

This study investigated the feasibility of visual and quantitative assessment of coronary vessel wall contrast enhancement (CE) for detection of symptomatic atherosclerotic coronary artery disease (CAD) and subclinical coronary vasculitis in autoimmune inflammatory disease (systemic lupus erythematosus [SLE]), as well as the association with aortic stiffness, an established marker of risk.

**Background:**

Coronary CE by cardiac magnetic resonance (CMR) is a novel noninvasive approach to visualize gadolinium contrast uptake within the coronary artery vessel wall.

**Methods:**

A total of 75 subjects (CAD: n = 25; SLE: n = 27; control: n = 23) underwent CMR imaging using a 3-T clinical scanner. Coronary arteries were visualized by a T2-prepared steady state free precession technique. Coronary wall CE was visualized using inversion-recovery T1 weighted gradient echo sequence 40 min after administration of 0.2 mmol/kg gadobutrol. Proximal coronary segments were visually examined for distribution of CE and quantified for contrast-to-noise ratio (CNR) and total CE area.

**Results:**

Coronary CE was prevalent in patients (93%, n = 42) with a diffuse pattern for SLE and a patchy/regional distribution in CAD patients. Compared with control subjects, CNR values and total CE area in patients with CAD and SLE were significantly higher (mean CNR: 3.9 ± 2.5 vs. 6.9 ± 2.5 vs. 6.8 ± 2.0, respectively; p < 0.001; total CE area: median 0.8 [interquartile range (IQR): 0.6 to 1.2] vs. 3.2 [IQR: 2.6 to 4.0] vs. 3.3 [IQR: 1.9 to 4.5], respectively; p < 0.001). Both measures were positively associated with aortic stiffness (CNR: r = 0.61, p < 0.01; total CE area: 0.36, p = 0.03), hypercholesterolemia (r = 0.68, p < 0.001; r = 0.61, p < 0.001) and hypertension (r = 0.40, p < 0.01; r = 0.32, p < 0.05).

**Conclusions:**

We demonstrate that quantification of coronary CE by CNR and total CE area is feasible for detection of subclinical and clinical uptake of gadolinium within the coronary vessel wall. Coronary vessel wall CE may become an instrumental novel direct marker of vessel wall injury and remodeling in subpopulations at risk.

Coronary contrast enhancement (CE) by cardiac magnetic resonance (CMR) is a novel, noninvasive approach for visualization of gadolinium contrast uptake within the coronary artery vessel wall. Previous studies demonstrated that CE colocalizes with mixed and calcified plaques in advanced coronary artery disease (CAD) (reviewed in Kuo et al. [1]). We have recently shown that coronary CE is also present in patients with systemic lupus erythematosus (SLE) despite the absence of cardiovascular (CV) symptoms (2). Demonstration of subclinical coronary involvement in subjects with persistent systemic inflammation, accelerated atherosclerosis, and increased and premature mortality and morbidity can be potentially useful for identification of individuals at risk 3, 4, 5.Younger women with SLE appear to be particularly affected; they experience an increased rate of coronary events early in the course of systemic disease (6). As current risk stratification schemes fail to detect these patients, a noninvasive and radiation-free approach using coronary CE may help to facilitate identification of early subclinical coronary vessel wall changes in vulnerable subpopulations (7). Patients with systemic inflammatory diseases, including SLE, are for the first time being acknowledged as subpopulations with increased CV risk in practice guidelines (8). Means of early and noninvasive identification of coronary vascular changes or strategies for improved prevention in these patients remain unknown. The aim of the current study was to assess the feasibility of coronary CE to describe differences between patients with established CAD and SLE, as well as control subjects. In addition to visualization, we employed 2 methods of CE quantification: contrast-to-noise ratio (CNR) and total CE area. We also determined the association of CE with aortic stiffness, an independent predictor of increased CV risk.

## Methods

A total of 25 patients with previously known significant CAD were included who had evidence of ≥50% stenosis on invasive coronary angiography or a previous myocardial infarction by late gadolinium enhancement (LGE). Twenty-seven subjects with an established diagnosis of SLE fulfilling the American College of Rheumatology revised classification criteria and with no previous history of cardiac symptoms were recruited from the Louise Coote Lupus Unit, St Thomas’ Hospital, London (9). The SLE disease activity was assessed using the SLE disease activity index score (10). Normotensive subjects (n = 23) with low pre-test likelihood for CV disease, taking no regular medication, with evidence of normal coronaries by either a previous invasive x-ray angiography or computed tomography (CT) coronary angiography, and consequently, with normal findings from CMR study, served as control subjects. A total of 12 SLE subjects and 6 control subjects that had concomitant acquisitions of coronary datasets as well as pulse wave velocity (PWV) for assessment of aortic stiffness were included in a previously published paper (2). Patient characteristics were recorded for all subjects, including age, sex, body mass index, renal function, presence of CV risk factors, and medication. Additional exclusion criteria were any general contraindication to contrast-enhanced or adenosine stress CMR. The study protocol was reviewed and approved by the local ethics committee, and written informed consent was obtained from all participants.

All patients underwent CMR for routine assessment of cardiac volumes and function and adenosine stress myocardial perfusion using a 3-T clinical scanner equipped with multitransmit technology and advanced cardiac software package (Achieva, Philips Healthcare, Best, the Netherlands). LGE imaging was performed approximately 20 min after an intravenous bolus of 0.2 mmol/kg of body weight of gadobutrol (Gadovist, Bayer Schering Pharma, Berlin, Germany) using complete short-axis stack coverage and long-axis views (2-, 3-, and 4-chamber views) (11). In-plane flow acquisitions of ascending and descending aorta and aortic arch were obtained for PWV measurement during shallow free-breathing, using a retrospectively gated gradient echo pulse sequence and signal averaging (for imaging parameters please see the Online Appendix) (12).

### Image analysis

All routine CMR analysis was performed using commercially-available software following standardized post-processing recommendations (13). Endocardial left ventricle (LV) borders were manually traced at end-diastole and -systole. The papillary muscles were included as part of the LV cavity volume. LV end-diastolic and -systolic volumes were determined using Simpson’s rule. Ejection fraction was computed as end-diastolic volume – (end-systolic volume/end-diastolic volume). All volumetric indexes were normalized to body surface area.

Short-axis LGE images were visually examined for the presence of regional fibrosis, which showed as bright areas within the myocardium in 2 fold-over directions and corresponding long-axis views and by exclusion of potential artifacts. Stress and rest perfusion images were evaluated visually for the pattern and presence of significant myocardial ischemia (13). Microvascular disease was established by circumferential homogeneous gradient of epicardial to endocardial enhancement (13). A space-averaged PWV was measured between ascending and thoracic descending aorta using an in-house developed software, as previously described 14, 15, 16.

### Coronary imaging technique and analysis

All coronary imaging studies were performed as free breathing scans with respiratory navigation (details on sequence parameters are provided in the Online Appendix). First, coronary arteries were localized by a 3-dimensional T2-prepared balanced steady-state free precession CMR angiography sequence (17). Double-oblique imaging planes parallel to the proximal RCA and LCA were then defined using a 3-point plan-scan tool for free-breathing targeted volume CMR angiography with a balanced steady-state free precession sequence with navigator-gating and correction 1, 2, 17. Coronary CE imaging was performed last using a T1-weighted 3-dimensional gradient echo inversion recovery sequence, typically 40 min after bolus administration, as previously described (18). Inversion time was determined by a prior Look-Locker sequence to optimize the contrast between brightness within the vessel wall due to gadolinium enhancement and suppressed coronary blood signal (typical time delay: 190 to 210 ms).

**Figure 1:**
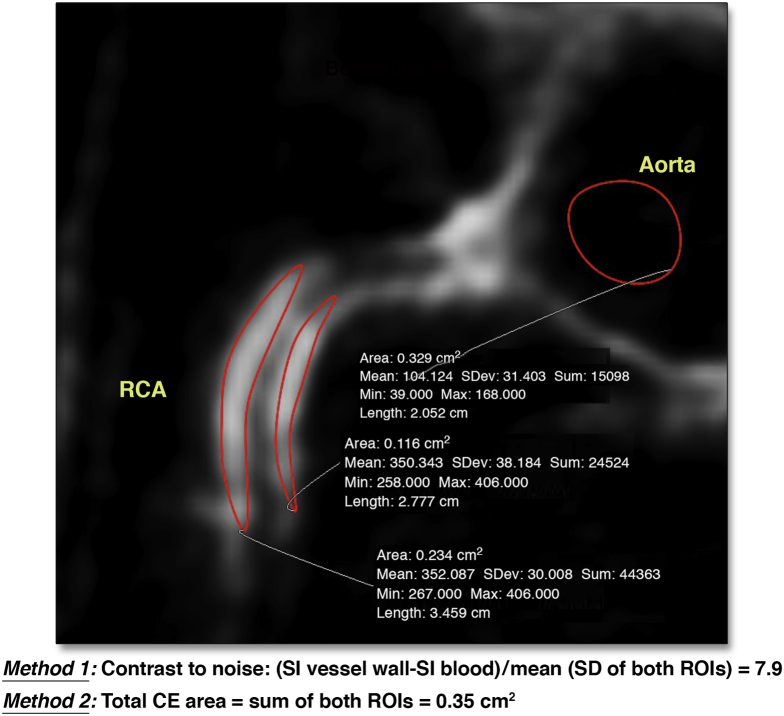
Quantification of Coronary CE Method 1—contrast to noise ratio (CNR)—is based on measuring signal intensities (SIs) of the vessel wall and blood pool. Method 2—total contrast enhancement (CE) area—is used to quantify the total area of enhancement. Calculation is performed for illustration in a patient with coronary artery disease. RCA = right coronary artery; ROI = region of interest.

Details of coronary CE post-processing are provided in the Online Appendix. In brief, 2 observers (N.V. and R.H.) with high intraobserver and interobserver reproducibility have analyzed all data in an independent manner, blinded to the results of invasive or CT angiography as well as to the type of subject. Datasets were evaluated visually for CE distribution and severity. CNR was calculated using measurements of signal intensity in the vessel wall and blood pool as previously described (Fig. 1, Online Fig. 1) 1, 2. Total CE area was measured by using nonenhancing parts of coronary vessels as reference (>2 SD in signal intensity above normal) using manual delineation of a region of interest (19).

### Statistical analysis

Normality of distributions was tested with the Kolmogorov-Smirnov statistic. Categorical data are expressed as percentages, and continuous variables as mean ± SD or median (interquartile range), as appropriate. Comparison of independent samples was performed using the Student *t* test, chi-square test, or 1-way analysis of variance and Kruskal-Wallis (Bonferroni post-hoc and Fisher exact tests for differences from controls, respectively), as appropriate for the type of the data. Intraobserver and interobserver reproducibility and agreement were performed using Bland-Altman methods. Associations were explored by simple linear and binary logistic regression analyses. Cut-off values for discrimination between health and disease were derived using receiver-operating characteristic curve analysis using the point that maximized the trade-off between specificity and sensitivity. All tests were 2-tailed, and a p value <0.05 was considered significant.

## Results

Patients with CAD were older than SLE patients and control subjects (p < 0.01) (Table 1). All groups had a predominance of females. CAD patients also had more traditional CV risk factors. SLE patients took disease-modifying agents (steroids [n = 25], mycophenolate [n = 17], hydroxychloroquine [n = 11], and warfarin [n = 12]), whereas both patient groups took renin-angiotensin system blockers (SLE: n = 14, 52%; CAD: n = 25, 100%), calcium-channel blockers (SLE: n = 5, 19%; CAD: n = 18, 72%), statins (SLE: n = 3, 11%; CAD: n = 25, 100%), and aspirin (SLE: n = 6, 22%; CAD: n = 25, 100%). SLE patients had low disease activity, with a median SLE disease activity index score of 0 (interquartile range: 0 to 3) (Table 1).

**Table 1:** Patient Characteristics

	Control Subjects (n = 23)	SLE (n = 27)	CAD (n = 25)	p Value
Age, yrs	44 ± 14	42 ± 16	59 ± 9^∗^	<0.01
Female	15 (64)	23 (85)†	16 (64)	0.02
BMI, kg/m^2^	26 ± 4	24 ± 5	28 ± 6	0.21
Heart rate, beats/min	64 ± 6	61 ± 7	58 ± 9	0.49
Systolic BP, mm Hg	123 ± 12	129 ± 19	139 ± 24^∗^	<0.01
Diastolic BP, mm Hg	81 ± 9	85 ± 14	98 ± 16^∗^	<0.01
Positive family history of CAD	2 (9)	3 (11)	8 (32)^∗^	<0.01
Smoking	3 (13)	5 (20)	10 (4)^∗^	<0.01
Hypercholesterolemia	3 (13)	5 (20)	24 (96)^∗^	<0.01
Diabetes	0 (0)	0 (0)	5 (23)	<0.01
SLEDAI	—	0 (0–3)	—	
History of lupus nephritis	—	12 (44)	—	
Antiphospholipid syndrome	—	12 (44)	—	
eGFR, ml/min	102 ± 7	82 ± 17†	78 ± 19^∗^	<0.01
C-reactive protein, mg/l	2.9 ± 1.2	5.1 ± 3.7†	4.9 ± 3.1^∗^	0.04
Erythrocytes sedimentation rate, mm/h	<7	39 ± 21†	—	

Values are mean ± SD or n (%). One-way analysis of variance and Kruskall-Wallis with post-hoc tests for differences ∗for controls vs. coronary artery disease (CAD) patients and †for controls vs. systemic lupus erythematosus (SLE).

BMI = body mass index; BP = blood pressure; CAD = coronary artery disease; ESR = erythrocyte sedimentation rate; eGFR = estimated glomerular filtration rate; SLE = systemic lupus erythematosus; SLEDAI = systemic lupus erythematosus disease activity index.

**Figure 2:**
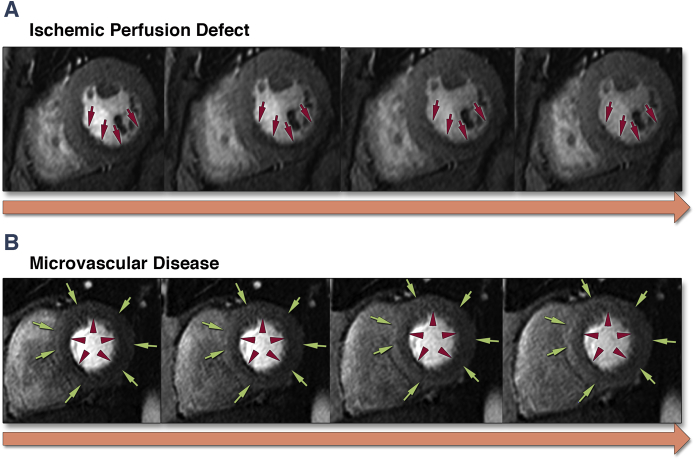
Representative Images of Myocardial Stress Perfusion Defects **(A)** Inducible ischemia, as evidenced by subendocardial perfusion defect in right coronary artery territory **(red arrows)**. **(B)** Circumferential homogeneous gradient demonstrating epicardial **(green arrows)** to endocardial **(red arrowheads)** enhancement.

Compared with control subjects and SLE patients, CAD patients had enlarged cardiac volumes, impaired global systolic function, and increased LV mass (Table 2). None of the patients had evidence of significant valvular pathology. Twenty-two CAD patients had inducible ischemia on adenosine myocardial perfusion testing, whereas 8 patients with SLE showed a pattern of microvascular disease (Fig. 2). Eleven SLE patients (41%) showed diffuse perimyocardial LGE, most commonly noted in midbasal inferolateral segments (20). Twenty CAD patients revealed an ischemic type of LGE (13). PWV was similarly significantly increased in both patient groups.

**Table 2:** Volumes and Function by Cardiac Magnetic Resonance

	Control Subjects (n = 23)	SLE (n = 27)	CAD (n = 25)	p Value
LVED index, ml/m^2^	79 ± 23	71 ± 18	112 ± 41^∗^	<0.001
LVES index, ml/m^2^	33 ± 11	39 ± 14	68 ± 49^∗^	<0.001
Ejection fraction, %	58 ± 7	56 ± 11	46 ± 18^∗^	0.02
LV mass index, g/m^2^	44 ± 21	49 ± 11	68 ± 31^∗^	0.05
Adenosine perfusion				
Abnormal	0 (0)	8 (30)	22 (88)	<0.001
Inducible ischemia	0 (0)	0 (0)	19 (76)	<0.001
Microvascular disease	0 (0)	8 (30)	0 (0)	<0.001
LGE, n present	0 (0)	11 (41)†	20 (80)^∗^	<0.01
Pulse wave velocity, m/s	4.7 ± 2.0	8.1 ± 3.1†	8.7 ± 2.6^∗^	<0.001
CE intensity				0.002
Mild	4 (17)	3 (11)	2 (8)	
Moderate	0 (0)	9 (33)	8 (32)	
Severe	0 (0)	15 (66)	15 (60)	
CE pattern				
Patchy/regional	3 (13)	3 (11)†	19 (76)^∗^	<0.001
Generalized	1 (4)	24 (89)†	6 (24)^∗^	<0.001
CNR	3.9 ± 2.5	6.9 ± 2.5†	6.8 ± 2.0^∗^	<0.001
Total area, mm^2^	0.8 (0.6–1.2)	3.2 (2.6–4.0)†	3.3 (1.9–4.5)^∗^	<0.001

Values are mean ± SD, n (%), or median (interquartile range). One-way ANOVA and Kruskall-Wallis with post-hoc tests for the differences ∗for controls vs. coronary artery disease (CAD) patients and †for controls vs. systemic lupus erythematosus (SLE).

CE = coronary enhancement; CNR = contrast to noise ratio; LGE = late gadolinium enhancement; LV = left ventricular; LVED = left ventricular end-diastolic; LVES = left ventricular end-systolic; other abbreviations as in Table 1.

### Coronary CE analysis

**Figure 3:**
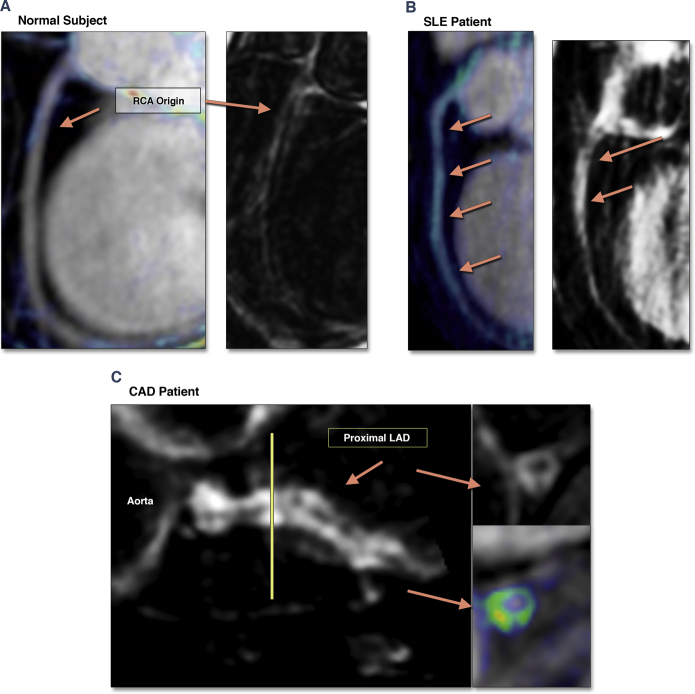
Representative Images of Clinical Findings **(A)** Control subject (female, age 38 years) with mild RCA enhancement **(blue). (B)** A patient with systemic lupus erythematosus (SLE) (female, age 36 years), generalized coronary enhancement over the projected long-axis view of the RCA. **(C)** A patient with coronary artery disease (CAD) (male, age 57 years), with patchy coronary enhancement within the area of soft plaque; short-axis view of the proximal left coronary artery. LAD = left anterior descending artery; other abbreviations as in Figure 1.

The proximal course of both coronary arteries (proximal to mid-segment) were visualized in all subjects and included in the visual assessment and quantification analysis of coronary CE. The image quality was rated as excellent (n = 25), good (n = 42), moderate (n = 7), and poor (n = 1). The majority of CAD patients (n = 21, 84%) had a previously known severe lesion (>75% stenosis) in at least 1 of the coronary territories, of which 11 patients had a total occlusion of the respective artery. Four control subjects showed mild coronary CE (Fig. 3A). Coronary CE was prevalent in both patient groups (Table 2). SLE patients showed a diffuse enhancement pattern (Fig. 3B), whereas in patients with CAD, CE was patchy and concentrated to the areas of minimal lumen diameter (Fig. 3C). Both patient groups had higher CNR and total CE area, with no difference between the patient groups.

### Analysis of relationships

CNR correlated with PWV and total CE area in the cohort as a whole and in separate groups (CNR controls: r = 0.51, p < 0.01; SLE: r = 0.69, p < 0.01; CAD: r = 0.64, p < 0.01) (Table 3). PWV and CNR were associated with age, but not with sex. All 3 vascular measures were associated with hypercholesterolemia and hypertension. Using binary logistic regression, both CE quantification measures were able to discriminate between control subjects and (all) patients (CNR [cut-off: 5.6]: sensitivity 96%, specificity 80%, accuracy 93% [hazard ratio: 6.1, 95% confidence interval: 2.4 to 10.8, p < 0.01], area under the curve: 0.95, p < 0.01; total CE area (cut-off: 1.2 mm^2^): sensitivity 87%, specificity 72%, accuracy 81% [hazard ratio: 2.9, 95% confidence interval: 1.3 to 4.4, p = 0.006], area under the curve: 0.82, p < 0.01). For more information about the reproducibility assessments, please see the Online Appendix.

**Table 3:** Results of Bivariate Regression Analyses

	CNR		Total CE area	
	r/rho	p Value	r/rho	p Value
CNR			0.43	0.01
Total CE area	0.43	<0.01		
Age	0.39	0.01	0.11	0.44
Sex	0.18	0.27	0.16	0.37
Heart rate	0.21	0.21	−0.13	0.31
Systolic BP	0.41	<0.01	0.32	0.01
Hypercholesterolemia	0.46	<0.01	0.39	0.01
Hypertension	0.40	<0.01	0.32	0.05
Diabetes	0.29	0.07	0.20	0.19
Pulse wave velocity	0.61	<0.01	0.36	0.03
C-reactive protein	0.38	0.05	0.29	0.09
Ejection fraction	−0.31	0.07	−0.27	0.11
LV-mass index	0.33	0.05	0.20	0.17
LGE (presence)	0.26	0.12	0.27	0.11

Correlations were performed using Pearson or Spearman tests, as appropriate for the type of the data.

Abbreviations as in Table 1, Table 2.

## Discussion

We demonstrate that quantification of coronary CE by CNR and total CE area provides meaningful values for discrimination between healthy subjects and patients with CAD and SLE. We also demonstrate that quantifiable measures of coronary CE are similar subclinically in patients with SLE and in patients with known and clinically-overt CAD, whereas the distribution of CE pattern is different. Both CE-CNR and total CE area are associated with PWV and increased cholesterol, established markers of increased CV risk. Our data suggest that visualization and quantification of coronary CE may provide a potential new marker of direct subclinical coronary remodeling and provide means to identify coronary involvement in vulnerable subgroups, such as SLE patients, ahead of symptomatic disease.

The standard approach to CV risk stratification relies on risk scores that target the benefit of population as a whole (8). It is recognized that, in many relevant subpopulations, CV risk is more compound and underestimated by these scores (e.g., young patients with comorbidities—systemic inflammatory diseases, impaired glucose tolerance, chronic kidney disease, and so on) 21, 22, 23. Imaging strategies of clinical practice rely on visualization of flow-limiting stenosis or more recently calcified plaque (invasive and CT coronary angiography, respectively) or detection of myocardial ischemia. Even though these methods provide prognostic information and guide therapy in symptomatic CV disease (24), their use as screening tools in young, vulnerable populations is limited. Repetitive radiation exposure in young patients is likely not justified despite considerable efforts to reduce radiation dose 25, 26. High calcium score correlates with more aggressive CV disease and reflects a greater burden in the general population (27). However, increased calcium score has poor correlation with disease activity or with the propensity of atherosclerotic plaques to rupture, making it difficult to predict acute coronary events (28). A previous study using calcium imaging in SLE patients showed that although age and body mass index predicted increased calcium score, SLE disease activity was not associated, challenging the utility of calcium scoring in this particular population (29). Whether calcium scoring is a useful marker of increased CV risk in young SLE patients has not been shown. Several studies reported abnormal myocardial perfusion in SLE patients; however, perfusion defects were not regional, as seen in CAD patients, but were more diffuse as typically seen in microvascular disease, adding to the pathophysiological distinction between the 2 conditions (30). Gold-standard methods for assessing coronary wall remodeling such as intravascular ultrasound or optical coherence tomography (19) are invasive and unsuitable for screening of young patients with recognized high risk.

As a noninvasive imaging strategy that is radiation-free and acceptable in most subjects, coronary CE imaging thus has the potential to develop into a novel marker to detect vulnerable individuals with subclinical coronary involvement. The physiological mechanism of gadolinium tissue uptake is well documented and corresponds with the extracellular space (31). CE imaging of the myocardium marks histological substrates such as scar, fibrosis, or extracellular edema 32, 33. Previous studies have shown that vessel wall CE colocalizes with areas of mixed and calcified plaque on CT. Coronary CE was also shown to be associated with acute or chronic systemic inflammation 1, 34. Validation studies performed in human vascular, mainly carotid, tissue suggest that gadolinium uptake in coronary vessels may bear similarities; however, direct histology, gadolinium quantification, and colocalization in the coronary vessel wall tissue is not available (35). In the absence of a direct histological correlate, we assume that findings in SLE patients correspond to subclinical coronary wall remodeling due to persistent systemic inflammation. A study in patients after cardiac transplantation lends support in showing that increased coronary CE corresponds to increased plaque burden, as demonstrated by intravascular ultrasound (19). Therefore, it might be possible to translate these findings to another vulnerable group. Although several studies have shown the feasibility of vessel wall CE imaging for the characterization of atherosclerotic disease (1), uptake of available gadolinium-based contrast agents is nonspecific and may represent acute and/or chronic inflammatory elements, such as edema, neovascularization, or fibrosis.

We demonstrate that coronary CE allows for discrimination between healthy subjects and patients with overt CAD and subclinical disease in SLE. We found that control subjects show relatively little contrast uptake and lower CNR values, whereas coronary CE is substantial (visually and quantifiably) in patients with CAD and SLE. The values of CNR and total CE area in both patient groups are comparable, despite the differences in age and distribution of the risk factors and clinical condition. We further show that CE pattern in SLE is predominantly diffuse, consistent with accelerated vessel remodeling, contrasting the patchy and regional involvement in CAD, which is more limited to the areas of focal plaque formation. Thus, qualitative assessment of coronary CE as present/absent and diffuse/patchy may allow for rapid discrimination between health/disease and type of disease, and is potentially of great clinical utility. Quantification of coronary CE may add a means to grade severity of disease.

The associations with increased aortic stiffness for the total cohort as well as for separate groups further support the concept that coronary CE concords with adverse vessel wall remodeling and may potentially provide a new and direct marker for individualized CV risk assessment in vulnerable subpopulations (21). Compared with previous studies reporting on coronary CE in patients with systemic inflammatory diseases 1, 34, 35, the prevalence of findings is higher in our study, and several reasons may explain this observation. Patient selection in the present study likely contributes most to this difference: patients with SLE have been recruited from a highly-specialized rheumatology service for complex systemic inflammatory diseases. It is conceivable that we included patients with more aggressive systemic disease, which is also reflected in their coronary involvement. Patients with CAD were similarly pre-selected as those with known and significant CAD. We also employed an optimized sequence with high spatial resolution at a higher field strength with greater signal-to-noise ratio (1). A sufficient time delay between contrast administration and post-contrast acquisition is crucial for maximized background tissue and blood suppression by inversion-recovery pre-pulse to allow for visualization of gadolinium uptake within the enhanced vessel wall, as previously reported (between 30 to 45 min after contrast administration) (18). Finally, we used gadobutrol, a gadolinium contrast agent with the highest magnetic “relaxivity,” allowing for the greatest shortening of T1 relaxation (and contrast) within accumulated tissues (36).

### Study limitations

This was an exploratory and hypothesis-generating pilot study with a limited sample size to test the feasibility of coronary CE imaging in pre-selected patient populations with a high CV risk. Thus, a few limitations apply to this study. We were not able to assess the true interstudy reproducibility (a repeated study on a different occasion). In a small number of patients, we repeated coronary acquisitions within the same study for various technical reasons; visualization of coronary CE or quantification measurements was not markedly different. We specifically focused on the proximal course of the coronary vessels, which is robust to visualization by CMR and allows excellent images of both the left and right coronary systems. Such an approach likely underlies the high reproducibility of CNR calculation/area measurement. Stents may pose an important limitation to future implementation of coronary CE as they obscure the insight into the vessel by inducing a void of magnetization, leading to an underestimation of CE area and obstructing the CNR calculation. In the present study, however, only a small number of CAD patients have had stents, and none of them were within the proximal segments. This is mainly explained by the nature of their presentation to CMR (upon which they were recruited to this study) for a test of myocardial ischemia to guide subsequent coronary intervention. Because we intend coronary CE as a means to identify the subclinical coronary injury early in the course of disease, we believe that it is unlikely that stents would be present in such patients and they would not pose a limitation to its use in practice. Associations with soluble biomarkers of disease and atherosclerotic plaque activity and the correlation with novel imaging techniques for characterization of atherosclerotic coronary plaque composition, such as coronary CT angiography, need to be examined in larger studies 19, 27, 35. Finally, the relation with poorer prognosis and ability to inform and guide the individualized risk assessment requires confirmation in future studies (37).

## Conclusions

Taken together, we demonstrate that visualization and quantification of coronary CE by CNR and total CE area may provide a new approach to the detection of subclinical and clinical coronary vessel wall remodeling and may support new ways to better characterize patients with SLE. Coronary CE may provide a potential new marker of individualized risk assessment in vulnerable subgroups.

## References

[1] Kuo Y.S., Kelle S., Lee C. (2014). Contrast-enhanced cardiovascular magnetic resonance imaging of coronary vessel wall: state of art. Expert Rev Cardiovasc Ther.

[2] Puntmann V.O., D’Cruz D., Taylor P.C. (2012). Contrast enhancement imaging in coronary arteries in SLE. J Am Coll Cardiol Img.

[3] van Leuven S.I., Franssen R., Kastelein J.J., Levi M., Stroes E.S., Tak P.P. (2008). Systemic inflammation as a risk factor for atherothrombosis. Rheumatology.

[4] Puntmann V.O., Taylor P.C., Mayr M. (2011). Coupling vascular and myocardial inflammatory injury into a common phenotype of cardiovascular dysfunction: systemic inflammation and aging—a mini-review. Gerontology.

[5] Ahmad Y., Shelmerdine J., Bodill H. (2007). Subclinical atherosclerosis in systemic lupus erythematosus (SLE): the relative contribution of classic risk factors and the lupus phenotype. Rheumatology.

[6] Bengtsson C., Ohman M.L., Nived O., Rantapää Dahlqvist S. (2012). Cardiovascular event in systemic lupus erythematosus in northern Sweden: incidence and predictors in a 7-year follow-up study. Lupus.

[7] Puntmann V.O., Bigalke B., Nagel E. (2010). Characterization of the inflammatory phenotype in atherosclerosis may contribute to the development of new therapeutic and preventative interventions. Trends Cardiovasc Med.

[8] Perk J., De Backer G., Gohlke H., European Association for Cardiovascular Prevention & Rehabilitation (EACPR), ESC Committee for Practice Guidelines (CPG) (2012). European guidelines on cardiovascular disease prevention in clinical practice (version 2012). The Fifth Joint Task Force of the European Society of Cardiology and Other Societies on Cardiovascular Disease Prevention in Clinical Practice (constituted by representatives of nine societies and by invited experts). Eur Heart J.

[9] Hochberg M.C. (1997). Updating the American College of Rheumatology revised criteria for classification of systemic lupus erythematosus. Arthritis Rheum.

[10] Gladman D., Ginzler E., Goldsmith C. (1996). The development and initial validation of the Systemic Lupus International Collaborating Clinics/American College of Rheumatology Damage Index for Systemic Lupus Erythematosus. Arthritis Rheum.

[11] Kramer C.M., Barkhausen J., Flamm S.D., Kim R.J., Nagel E., Society for Cardiovascular Magnetic Resonance Board of Trustees Task Force on Standardized Protocols (2008). Standardized cardiovascular magnetic resonance imaging (CMR) protocols, Society for Cardiovascular Magnetic Resonance: Board of Trustees Task Force on Standardized Protocols. J Cardiovasc Magn Reson.

[12] Rogers W.J., Hu Y.L., Coast D., Vido D.A., Kramer C.M., Pyeritz R.E., Reichek N. (2001). Age-associated changes in regional aortic pulse wave velocity. J Am Coll Cardiol.

[13] Schulz-Menger J., Bluemke D.A., Bremerich J. (2013). Standardized image interpretation and post processing in cardiovascular magnetic resonance: Society for Cardiovascular Magnetic Resonance (SCMR) Board of Trustees Task Force on Standardized Post Processing. J Cardiovasc Magn Reson.

[14] Puntmann V.O., Nagel E., Hughes A.D. (2012). Gender-specific differences in myocardial deformation and aortic stiffness at rest and dobutamine stress. Hypertension.

[15] Gaddum N.R., Alastruey J., Beerbaum P., Chowienczyk P., Schaeffter T. (2013). A technical assessment of pulse wave velocity algorithms applied to non-invasive waveforms. Annals of Biomedical Engineering.

[16] Laurent S., Cockcroft J., Van Bortel L. (2006). Expert consensus document on arterial stiffness: methodological issues and clinical applications. Eur Heart J.

[17] Botnar R.M., Stuber M., Danias P.G., Kissinger K.V., Manning W.J. (1999). Improved coronary artery definition with T2-weighted, free-breathing, three-dimensional coronary MRA. Circulation.

[18] Kelle S., Schlendorf K., Hirsch G.A. (2010). Gadolinium enhanced MR coronary vessel wall imaging at 3.0 Tesla. Cardiol Res Pract.

[19] Hussain T., Fenton M., Peel S.A. (2013). Detection and grading of coronary allograft vasculopathy in children with contrast-enhanced magnetic resonance imaging of the coronary vessel wall. Circ Cardiovasc Imaging.

[20] Puntmann V.O., D’Cruz D., Smith Z. (2013). Native myocardial T1 mapping by cardiovascular magnetic resonance imaging in subclinical cardiomyopathy in patients with systemic lupus erythematosus. Circ Cardiovasc Imaging.

[21] Esdaile J.M., Abrahamowicz M., Grodzicky T. (2001). Traditional Framingham risk factors fail to fully account for accelerate atherosclerosis in systemic lupus erythematosus. Arthritis Rheum.

[22] Cruickshank K., Riste L., Anderson S.G., Wright J.S., Dunn G., Gosling R.G. (2002). Aortic pulse-wave velocity and its relationship to mortality in diabetes and glucose intolerance: an integrated index of vascular function?. Circulation.

[23] Schiffrin E.L., Lipman M.L., Mann J.F. (2007). Chronic kidney disease: effects on the cardiovascular system. Circulation.

[24] Gibbons R.J., Abrams J., Chatterjee K. (2003). ACC/AHA 2002 guideline update for the management of patients with chronic stable angina—summary article: a report of the American College of Cardiology/American Heart Association Task Force on Practice Guidelines (Committee on the Management of Patients With Chronic Stable Angina). J Am Coll Cardiol.

[25] van Werkhoven J.M., Gaemperli O., Schuijf J.D. (2009). Multislice computed tomography coronary angiography for risk stratification in patients with an intermediate pretest likelihood. Heart.

[26] Einstein A.J., Henzlova M.J., Rajagopalan S. (2007). Estimating risk of cancer associated with radiation exposure from 64-slice computed tomography coronary angiography. JAMA.

[27] Polonsky T.S., McClelland R.L., Jorgensen N.W. (2010). Coronary artery calcium score and risk classification for coronary heart disease prediction. JAMA.

[28] Muntendam P., McCall C., Sanz J., Falk E., Fuster V., High-Risk Plaque Initiative (2010). The BioImage Study: novel approaches to risk assessment in the primary prevention of atherosclerotic cardiovascular disease–study design and objectives. Am Heart J.

[29] Kiani A.N., Magder L., Petri M. (2008). Coronary calcium in systemic lupus erythematosus is associated with traditional cardiovascular risk factors, but not with disease activity. J Rheumatol.

[30] Ishimori M.L., Martin R., Berman D.S. (2011). Myocardial ischemia in the absence of obstructive coronary artery disease in systemic lupus erythematosus. J Am Coll Cardiol Img.

[31] Rehwald W.G., Fieno D.S., Chen E.L., Kim R.J., Judd R.M. (2002). Myocardial magnetic resonance imaging contrast agent concentrations after reversible and irreversible ischemic injury. Circulation.

[32] Kim R.J., Wu E., Rafael A. (2000). The use of contrast-enhanced magnetic resonance imaging to identify reversible myocardial dysfunction. N Engl J Med.

[33] Friedrich M.G., Sechtem U., Schulz-Menger J., International Consensus Group on Cardiovascular Magnetic Resonance in Myocarditis (2009). Cardiovascular magnetic resonance in myocarditis: a JACC white paper. J Am Coll Cardiol.

[34] Schneeweis C., Schnackenburg B., Stuber M. (2012). Delayed contrast-enhanced MRI of the coronary artery wall in takayasu arteritis. PloS One.

[35] McAteer M.A., Akhtar A.M., von Zur Muhlen C., Choudhury R.P. (2010). An approach to molecular imaging of atherosclerosis, thrombosis, and vascular inflammation using microparticles of iron oxide. Atherosclerosis.

[36] Rohrer M., Bauer H., Mintorovitch J., Requardt M., Weinmann H.J. (2005). Comparison of magnetic properties of MRI contrast media solutions at different magnetic field strengths. Invest Radiol.

[37] Puntmann V.O. (2009). How-to guide on biomarkers: biomarker definitions, validation and applications with examples from cardiovascular disease. Postgrad Med J.

