# Supplementary material for: Coronary Vessel Wall Contrast Enhancement Imaging as a Potential Direct Marker of Coronary Involvement: Integration of Findings From CAD and SLE Patients
Source: JACC Cardiovasc Imaging. 2014 Aug;7(8):762–70. doi: 10.1016/j.jcmg.2014.03.012 (PMC4136741; doi:10.1016/j.jcmg.2014.03.012)
Supplement: Online Appendix and Online Figure 1 [file mmc1.docx]

**Supplementary material**

**Methods - Sequence parameters**

**Inplane phase contrast gradient echo sequence for acquisition of PWV**

In-plane flow acquisitions of ascending and descending aorta and aortic arch were obtained for PWV measurement during shallow free-breathing, using a retrospectively gated gradient echo pulse sequence and signal averaging. Imaging parameters included TR/TE/flip-angle: 3.7ms/2.2ms/15°; spatial resolution (acquired): 2.3 mm x 2.3 mm x 10 mm; velocity encoding 200 cm/s, heart phase duration 6.2 msec, 120 acquired phases and all scans were acquired in sagittal oblique views, as previously described [**12**].

**Coronary imaging studies**

Coronary studies were performed at the end of the clinical protocol after myocardial LGE imaging to allow for a sufficient time delay for accumulation of gadolinium in the vessel wall and washout of contrast agent from the blood pool.

1. Whole heart coronary scout 3-dimensional (3D) T2-prepared balanced steady-state free precession (SSFP) coronary MR angiography (CMRA) sequence with acquisition in transverse orientation (imaging parameters included field of view (FOV)=320x320 mm, acquired in-plane resolution=1.45x1.45 mm, slice thickness=1.9mm, acquisition window=80-100ms, repetition time (TR)/echo time(TE)/flip angel (FA) 4.8msec/2.2msec/70°, and number of slices=90-120). Typical imaging time = 2-4 minutes.
2. Targeted volume single coronary artery acquisitions using a 3-point plan-scan tool for free-breathing targeted volume CMRA with a balanced SSFP sequence (imaging parameters included field of view (FOV)=320x320 mm, acquired in-plane resolution=1.25x1.25 mm, slice thickness=3mm, acquisition window=80-100ms, repetition time (TR)/echo time(TE)/flip angel (FA) 5.3 msec/1.5msec/20°, and number of slices=21). Typical acquisition time = 1.5-2 minutes.
3. Coronary CE imaging was performed last using a T1-weighted 3D gradient echo inversion recovery sequence typically 40 minutes after bolus administration, as previously described [**18]**. Imaging parameters included FOV: 320x320 mm, acquired in-plane resolution=1.25x1.25 mm, slice thickness=3mm, TR/TE/FA=5.7 msec/1.7 msec/30°, a non-selective adiabatic inversion recovery prepulse instead of T2 preparation for magnetization preparation, and number of slices=21). Typical acquisition time = 1.5-2 minutes.

**Coronary imaging postprocessing analysis**

Coronary CE was defined as areas in the vessel wall displaying comparable brightness to the ascending aortic vessel wall within the same imaging slice. Coronary CE of proximal segments was visually classified for distribution (as patchy/regional or diffuse) and its severity (trace - barely visible, mild - covering less than 10% of the proximal segment, moderate ≤ 50%, severe >50%). Coronary wall SI was assessed by placing a region-of-interest (ROI) tightly within the visually enhanced area within proximal segments (**Figure 1**). Coronary blood SI (SI blood) was determined from ROI within the blood pool of ascending aorta within the same imaging slice as the coronary wall SI to ensure similar image scaling. Coronary wall CNR was calculated as follows: CNR=(SIwall-SIblood)/SD noise [**1,2**]. Noise was calculated as a mean of SD of within the two ROIs (wall and blood pool). In subjects, where both proximal coronary systems were visible, CNR and area of enhancement measurements were obtained from both RCA and LCA, and derived as a mean of both measurements for a single subject. The assessment of total CE area has been performed by modification of a previously described method [**1,19**]. Total CE area was measured by using non-enhancing parts of coronary vessels as reference (> 2SD in SI above the normal)**.** The area of coronary CE was then quantified by manual delineation of a ROI [**Figure 1**]. The two observers graded the image quality for whether diagnostic, and the severity of artefacts. Coronary CE CNR and total area measurements were repeated to calculate the intraobserver and interobserver variabilities.

**Results**

**Reproducibility assessment (supplementary material).**

Inter and intra-observer reproducibility of CNR quantification (**Supplemental Figure 1A**) was excellent (intra: r=0.97; P<0.01; mean difference (MD)±SD=-0.25±0.43; inter: r=0.95, P<0.01; MD±SD=-0.28±0.51). Assessment of total CE area also showed a good agreement (**Supplemental Figure 1B**) (intra: r=0.90, p<0.01, MD±SD (mm2)= 0.17±0.97); inter: r=0.88, p<0.05, MD±SD (mm2)=-0.28±0.84). A repeated coronary acquisition within the same imaging study (n=7) served for interstudy reproducibility assessment showing reasonable reproducibility and agreement MD±SD=0.41±0.74, r=0.86, P<0.01.

**Supplemental Figure 1. Bland Altman plots for assessment of intra- and interobserver reproducibility.** **A**- CNR, **B**-total CE area**.**
